# Enhanced recovery after surgery (ERAS) in elderly patients undergoing high-complexity colorectal surgery: a retrospective cohort study from a Latin American tertiary center

**DOI:** 10.1097/MS9.0000000000005745

**Published:** 2026-10-22

**Authors:** Jessica Capre-Pereira, Helber-Fabián Pérez-Imbachí, Juan-Guillermo Holguín, Eduardo Posada-Angel, Andrés Gempeler, Einar Billefals, Mauricio Burbano, Valentina Aguirre, Estefanía Gallego, Abraham Kestenberg

**Affiliations:** a División de Cirugía Colorrectal, Fundación Valle del Lili, Cali, Colombia; b Facultad de Ciencias de la Salud, Universidad ICESI, Cali, Colombia; c Centro de Investigaciones Clínicas, Fundación Valle del Lili, Cali, Colombia; d Capítulo de Futuros Cirujanos, Asociación Colombiana de Cirugía, Bogotá, Colombia; e Departamento de Anestesiología, Fundación Valle del Lili, Cali, Colombia

**Keywords:** aged, colorectal surgery, enhanced recovery after surgery, outcomes

## Abstract

**Background:**

Enhanced recovery after surgery (ERAS) protocols have been shown to improve perioperative outcomes in colorectal surgery; however, evidence focusing specifically on elderly patients undergoing high-complexity resections remains limited. This study assessed the association of ERAS implementation among patients aged 65 years and older undergoing elective major colorectal surgery.

**Methods:**

A single-center cohort study using a historical control design was conducted between January 2018 and January 2026. Elderly patients (defined as ≥65 years) undergoing elective high-complexity colorectal surgery were included. Exclusion criteria included synchronous liver metastasis resection, urgent/emergency surgery, or a World Health Organization performance status of 4. The primary outcome was overall postoperative complications within 30 days after surgery. The risk analysis of the outcomes was performed using crude and adjusted risk ratios (aRR) with robust Poisson regression, adjusted for demographic and clinical confounders.

**Results:**

A total of 192 patients were included (non-ERAS: 83; ERAS: 109). The median age was 74 years [interquartile ranges (IQR): 70–79], and 52.6% were women. The most common procedures were anterior rectal resection (39.6%) and right hemicolectomy (29.7%). ERAS patients had a significantly shorter hospital stay [3 days (IQR: 2–5) vs. 4 days (IQR: 3–9); *P* = 0.002]. An adjusted relative risk reduction for overall postoperative complications of 59% [aRR = 0.41, 95% confidence intervals (CI) = 0.27–0.63, *P* < 0.001] was found. Adjusted relative risk reductions were also found for unplanned intensive care admission, readmissions, and reinterventions.

**Conclusion:**

ERAS implementation in elderly patients undergoing high-complexity colorectal surgery was associated with lower postoperative morbidity, intensive care unit admission rates, and hospital length of stay, without any documented adverse outcomes.

## Introduction

Colorectal surgery is associated with substantial postoperative morbidity, a burden that is disproportionately higher among older adults. Despite advances in surgical technique and perioperative care, high complication rates after colon and rectal procedures persist^[^1,2^]^. As the global population aged 65 years or older continues to grow, surgeons increasingly treat patients with reduced physiological reserve and multiple comorbidities, which heighten vulnerability to postoperative complications and delayed recovery^[^3,4,5^]^. Beyond chronological age, frailty (manifested by sarcopenia, malnutrition, and impaired functional status) has emerged as a key determinant of adverse postoperative outcomes in this population^[^6^]^.

HIGHLIGHTS

- This study evaluates the clinical impact of enhanced recovery after surgery (ERAS) implementation in elderly patients undergoing high-complexity colorectal surgery at a Latin American center.
- ERAS implementation was associated with a shorter hospital stay and significant reductions in overall postoperative complications.
- Lower risks of unplanned intensive care unit admissions, readmissions, and reinterventions were observed among ERAS patients.
- Benefits were achieved without an increase in postoperative adverse outcomes.
- This study provides novel real-world evidence from Latin America, where data on ERAS outcomes in elderly patients undergoing complex colorectal surgery are scarce.

In parallel, enhanced recovery after surgery (ERAS) protocols have fundamentally reshaped perioperative care across multiple surgical disciplines. In colorectal surgery, previous evidence shows that ERAS implementation is associated with lower postoperative morbidity and faster recovery^[^7^]^. Meta-analyses from diverse healthcare settings consistently show that ERAS protocols reduce hospital length of stay by approximately 2.5 days compared with conventional care and are associated with lower postoperative morbidity, with reported rates ranging from 4 to 47%, whereas conventional management is associated with morbidity rates between 8 and 75%^[^8,9,10,11^]^.

Despite these well-established benefits, evidence specifically addressing the impact of ERAS in elderly patients undergoing high-complexity colorectal surgery remains limited. Furthermore, data from middle-income countries and Latin American healthcare systems are scarce; differences in resource availability, intensive care unit (ICU) capacity, and perioperative care organization may influence both the implementation and effectiveness of ERAS pathways. Therefore, this study aimed to evaluate the association between ERAS protocol implementation and 30-day postoperative outcomes in patients aged 65 years and older undergoing elective high-complexity colorectal surgery at a tertiary referral center. This study has been reported in line with the STROCSS guidelines^[^12^]^.

## Methods

### Study design and setting

A retrospective cohort study with historical controls was conducted using medical records from January 2018 to January 2026 at a high-complexity tertiary-referral university hospital in Southwestern Colombia. A historical control approach was selected due to the institutional, service-wide implementation of ERAS, which precluded contemporaneous randomization.

The study was designed to emulate a pragmatic clinical experiment evaluating the effect of ERAS implementation in routine clinical practice^[^13^]^.

### Population

Adult patients aged 65 years or older undergoing elective major colorectal surgery for benign or malignant disease were eligible for inclusion. The age threshold used to define elderly patients in this study was based on consensus statements and prior publications that classify individuals aged 65 years and older as older adults^[^14,15^]^. Major, high-complexity colorectal surgery was defined as segmental or total colonic or rectal resection, with or without anastomosis, requiring general anesthesia, intra-abdominal dissection, a projected operative time of 2 hours or longer, and planned inpatient postoperative care at a tertiary referral center. Emergency procedures, synchronous liver metastasis resections, and patients with severe preoperative functional limitation (World Health Organization performance status ≥ 4) were excluded^[^16^]^.

### Conventional management

During the historical control period (January 2018 to October 2022), patients received conventional perioperative care directed by the treating colorectal surgeon, who performed the preoperative assessment and prescribed perioperative orders. In this group, as in the ERAS group, all patients underwent a comprehensive geriatric assessment prior to surgery to determine surgical fitness and appropriateness for operative management. Conventional care did not include preoperative carbohydrate loading, and bowel preparation and fasting were routinely performed the day before surgery. Intraoperatively, anesthetic management (including fluid administration, hemodynamic monitoring, and vasoactive or transfusion support) was provided according to standard practice. Intraoperative fluid administration was generally based on liberal crystalloid replacement strategies at the discretion of the anesthesiology team, without standardized goal-directed fluid therapy protocols. Maintenance intravenous fluids were commonly continued until the day prior to hospital discharge or until complete oral tolerance was achieved, defined as tolerance of a full solid diet. Prophylactic antiemetics and scheduled multimodal analgesia were not routinely administered. Removal of nasogastric and urinary catheters was left to the discretion of the attending physician, and postoperative medication adjustments were made during daily ward rounds. Decisions regarding mobilization, initiation of oral intake, and hospital discharge were guided by the patient’s clinical course. Outpatient follow-up was typically scheduled 2–3 weeks after surgery.

### ERAS protocol

The ERAS protocol was approved by the institution in October 2022 and subsequently certified by the ERAS® Society in November 2023. As in the historical control group, all patients underwent a comprehensive geriatric assessment prior to surgery to determine surgical fitness. ERAS implementation relied on close coordination among multidisciplinary teams. The colorectal surgery team led preoperative evaluation, prioritized minimally invasive approaches when feasible, and directed postoperative management, including medication prescriptions, drain removal, advancement of oral intake, and discharge planning. The anesthesiology team was responsible for perioperative risk assessment, anemia optimization, anesthetic management, and postoperative pain control, including the use of epidural analgesia when indicated. Removal of urinary catheters was jointly coordinated by surgeons and anesthesiologists.

The nursing team provided structured patient education, coordinated preoperative preparation, and closely monitored postoperative recovery, including dietary progression and pain control, with follow-up telephone calls on days 15 and 30 days. The nutrition team performed individualized nutritional assessments and delivered perioperative nutritional support. The physical therapy team implemented prehabilitation strategies, early mobilization, and supervised respiratory exercises starting on the day of surgery.

### Variables and clinical outcomes

Clinical and surgical variables were collected for all patients, including age, sex, medical history, substance use history, primary diagnosis, type of surgical intervention, and American Society of Anesthesiologists (ASA) physical status classification. Intraoperative variables included the specific procedure performed, operative findings, creation of an anastomosis or stoma, estimated blood loss, and the need for hemodynamic support or blood transfusion.

The primary outcome of this study was the occurrence of overall postoperative complications within 30 days after surgery. A postoperative complication was defined as any unplanned event during the postoperative period that adversely affected the patient’s health status^[^17,18^]^. Complications were classified as medical or surgical and included events such as anastomotic dehiscence, urinary tract injury, mechanical bowel obstruction, postoperative paralytic ileus, deep wound dehiscence, and hematoma.

Secondary outcomes included unplanned ICU admission (unexpected transfer to the ICU occurring after completion of surgery in a patient who was not scheduled for planned postoperative ICU care due to the development of medical or surgical complications or the need for advanced hemodynamic, respiratory, or organ support), primary hospital stay (the number of days from the date of surgery to the date of hospital discharge during the index hospitalization), tolerance to oral intake (the number of days from the date of surgery to the first documented tolerance of solid or semi-solid diet without nausea, vomiting, or need for nasogastric decompression), readmissions (any unplanned hospital readmission), and reoperations (unplanned return to the operating room). All outcomes were assessed over a 30 day postoperative period. Patients were analyzed according to their assigned study group, regardless of their level of adherence to individual ERAS protocol components, to ensure complete inclusion of all eligible individuals for group comparison.

### Sample size

All consecutive eligible patients during the study period were included in the analysis; therefore, a convenience sampling strategy was applied. A post hoc sample size calculation was subsequently performed based on the effect estimates observed in the previous analysis of the study protocol^[^19^]^. Assuming a control-group postoperative complication rate of 50.8% and an expected adjusted relative risk of 0.43, with a two-sided alpha of 0.05, 90% power, and a 1:1 allocation ratio, a minimum of 56 patients per group (112 total) would be required. After accounting for an anticipated 15% rate of missing data or losses, the estimated required sample size increased to approximately 66 patients per group (132 total).

### Bias

To reduce selection bias, uniform eligibility criteria were applied across both study periods, and group allocation was determined exclusively by the timing of ERAS implementation rather than by investigator discretion. Although the use of historical controls may introduce temporal bias, both cohorts were treated at a high-complexity institution with consistent infrastructure, staffing, and perioperative care standards. Clinical data were extracted from electronic medical records using predefined variables and standardized collection procedures to minimize information bias.

Additional safeguards were implemented to address limitations inherent to the observational design. These included a strict operational definition of high-complexity surgery, structured and uniform data abstraction, and multivariable regression analyses to adjust for baseline imbalances. Notably, the colorectal surgery and anesthesiology teams remained stable throughout the study period, reducing the likelihood that observed differences were attributable to changes in provider expertise or institutional practice patterns.

### Statistical analysis

Database closure and statistical analyses were performed only after all included patients had completed the predefined 30 day postoperative follow-up period. Quantitative variables were summarized using medians and interquartile ranges (IQRs) given their non-normal distribution and compared between groups using the Mann–Whitney U test. Categorical variables were expressed as absolute frequencies and percentages and compared using the chi-square test or Fisher’s exact test, as appropriate.

To estimate the association between ERAS implementation and clinical outcomes, relative risks (RRs) with 95% confidence intervals (CIs) were calculated. For multivariable analysis, robust Poisson regression models were fitted to estimate adjusted risk ratios (aRRs) and to account for potential confounding^[^18,19,20^]^. Covariates were selected *a priori* based on clinical relevance and included age, sex, body mass index, ASA classification, preoperative chemotherapy or radiotherapy, nutritional status, comorbid cardiovascular or pulmonary disease, smoking status, and procedure length. Nutritional status was incorporated into multivariable analyses using an explicit “Not evaluated” category to avoid excluding a substantial number of observations and to account for differences in nutritional assessment documentation between study periods. A purposeful selection strategy was used to optimize confounding control while minimizing model overfitting. Results are reported as RRs with 95% CIs, and statistical significance was defined as a two-tailed *P* < 0.05.

## Results

### Sociodemographic and clinical characteristics

During the study period, a total of 416 patients underwent major colorectal surgery, of whom 192 were who were aged 65 years or older and constituted the study population. Of these, 83 patients were managed under conventional care and 109 under the ERAS protocol. The median age was 74 years (IQR: 70–79), and 52.6% (101/192) were women. On perioperative assessment, 65.6% (126/192) were classified as ASA physical status II. The most frequently performed procedure was anterior rectal resection (39.6%, 76/192), followed by right hemicolectomy (29.7%, 57/192). The most common diagnosis was primary adenocarcinoma, present in 76.0% (146/192) of patients. No statistically significant differences were identified between groups regarding age, BMI, comorbidities, type of surgery, or final diagnosis. Baseline differences were observed in smoking history (*P* = 0.001), ASA classification (*P* = 0.018), and nutritional status assessment (*P* = 0.001). Additional sociodemographic and clinical characteristics are presented in Table 1.

**Table 1:** Clinical and demographic characteristics.

	Non-ERAS 83	ERAS 109
Age, median (IQR)	74 (69–81.5)	73 (70–78)
BMI, median (IQR)	24.42 (22.36–27.02)	24.23 (22.09–26.53)
Nutritional status		
Not evaluated	50 (60.2)	0 (0.0)
Normal status	24 (28.9)	82 (75.2)
Risk of malnutrition	2 (2.4)	17 (15.6)
Malnutrition	7 (8.4)	10 (9.2)
Smoker, (%)	12 (14.5)	2 (1.8)
Diabetes mellitus, (%)	20 (24.1)	27 (24.8)
Pulmonary disease, (%)	4 (4.8)	10 (9.2)
Cardiovascular disease, (%)	13 (15.7)	18 (16.5)
ASA classification, (%)		
ASA I	1 (1.2)	0 (0.0)
ASA II	46 (55.4)	80 (73.4)
ASA III	33 (39.8)	29 (26.6)
ASA IV	3 (3.6)	0 (0.0)
Type of surgery, (%)		
Right hemicolectomy	22 (26.5)	35 (32.1)
Left hemicolectomy	6 (7.2)	7 (6.4)
Anterior rectal resection	36 (43.4)	40 (36.7)
Hartmann procedure closure	3 (3.6)	12 (11.0)
Abdominoperineal resection	3 (3.6)	6 (5.5)
Other procedures	13 (15.7)	7 (6.4)
Final diagnosis, (%)		
Primary adenocarcinoma and other primary neoplasm	64 (77.1)	88 (80.7)
Benign tumor (including polyps)	3 (3.6)	3 (2.8)
Diverticular disease	7 (8.4)	8 (7.3)
Other diseases	9 (10.8)	10 (9.2)

### Adherence of ERAS protocol

Overall adherence to ERAS protocol components was significantly higher in the ERAS group, with a median overall adherence of 80.0% (IQR: 73.91–85.19) compared with 44.83% (IQR: 40.74–50.00) in the non-ERAS group (*P* < 0.001). Adherence was highest during the preadmission and preoperative phases, reaching 100% in the ERAS patients, whereas postoperative adherence, although lower, was significantly higher than in the conventional care group (58.33% vs. 25.00%, *P* < 0.001). Among postoperative ERAS components, same-day postoperative mobilization had the lowest adherence, particularly during the initial phase of protocol implementation, mainly due to resistance from family members and ward nursing staff regarding early mobilization in elderly postoperative patients. Intraoperative adherence was high in both groups.

### 30-day clinical outcomes

Overall, 30 day postoperative outcomes are summarized in Table 2. The median primary hospital length of stay for the entire cohort was 3 days (IQR: 2–7). Patients managed with the ERAS protocol had a significantly shorter length of stay compared with those receiving conventional care [3 days (IQR: 2–5) vs. 4 days (IQR: 3–9), *P* = 0.002]. Primary anastomosis was achieved in 83.3% (160/192) of patients, with no significant differences between groups (*P* = 0.068). There were also no significant between-group differences in postoperative nutritional support requirements (*P* = 0.190) or time to tolerating oral intake (*P* = 0.193).

**Table 2:** Clinical outcomes at 30 postoperative days.

	Non-ERAS 83	ERAS 109	*P*-value^a^
Overall complications, (%)	45 (54.2)	29 (26.6)	<0.001
In-hospital complications, (%)	38 (45.8)	20 (18.3)	<0.001
ICU admissions, (%)	31 (37.3)	20 (18.3)	0.005
Nutritional support, (%)			0.190
Not required	67 (80.7)	97 (89.0)	
Oral supplements	2 (2.4)	0 (0.0)	
Enteral nutrition	1 (1.2)	0 (0.0)	
Enteral and parenteral nutrition	3 (3.6)	1 (0.9)	
Parenteral nutrition	10 (12.0)	11 (10.1)	
Reinterventions, (%)	12 (14.5)	5 (4.6)	0.033
Cause of reinterventions, (%)			0.096
Anastomotic leakage	5 (6.0)	1 (0.9)	
Intra-abdominal Abscess	1 (1.2)	0 (0.0)	
Hemorrhage	0 (0.9)	1 (0.9)	
Wound dehiscence	2 (2.4)	2 (1.8)	
Other	4 (4.8)	1 (0.9)	
Oral tolerance, median (IQR)	10 (8–29)	9 (7.5–13)	0.193
Readmissions, (%)	15 (18.1)	10 (9.2)	0.110
Length of primary Hospital stay, median (IQR)	4 (3–9)	3 (2–5)	0.002
Length of total hospital stay, median (IQR)	5 (3–13)	3 (2–6)	0.002

^a^ Mann–Whitney U test, Chi2/Fisher test.

Overall, postoperative complications occurred in 38.5% (74/192) of patients. Complications were significantly more frequent in the non-ERAS group than in the ERAS group (54.2% vs. 26.6%, *P* < 0.001), with a comparable reduction observed for in-hospital complications (45.8% vs. 18.3%, *P* < 0.001). Paralytic ileus was the most common complication (11.5%, 22/192), with no significant difference between groups (*P* = 0.363). Similarly, rates of anastomotic leakage did not differ significantly (*P* = 0.276). Overall, the distribution of Clavien–Dindo grades differed significantly between groups, with ERAS patients experiencing less severe complications (*P* = 0.004). Thirty-day postoperative mortality was 0.5% (1/192).

### Unadjusted and adjusted risk analysis

Unadjusted and adjusted relative risk estimates accounting for baseline and clinical variables are presented in Table 3. In unadjusted analyses, ERAS implementation was associated with a 51% relative risk reduction in overall postoperative complications, a 51% reduction in unplanned ICU admission, and a 68% reduction in reinterventions. After adjustment, ERAS implementation remained independently associated with lower risks of overall postoperative complications, ICU admission, readmissions, and reinterventions. Although the reduction in 30 day readmissions did not reach statistical significance in the unadjusted analysis, this association became statistically significant after multivariable adjustment. The magnitude and direction of the associations were preserved after adjustment for potential confounders.

**Table 3:** unadjusted and adjusted risk analysis for clinical outcomes at 30 postoperative days.

Variable	Risk ratio	95% CI	*P*-value	Adjusted risk ratio^a^	95% CI	*P*-value
Complications	0.49	0.34–0.71	<0.001	0.41	0.27–0.63	<0.001
ICU admission	0.49	0.30–0.80	0.003	0.41	0.23–0.72	0.002
Readmission	0.51	0.24–1.07	0.06	0.44	0.20–0.97	0.04
Reinterventions	0.32	0.12–0.87	0.017	0.17	0.04–0.72	0.02

^a^ Models adjusted by age, body mass index, preoperative chemotherapy/radiotherapy, ASA classification, nutritional status, smoking, pulmonary/cardiovascular disease, and procedure length.

## Discussion

In this analysis, ERAS implementation was associated with a shorter hospital length of stay and a relative risk reduction of 30 day postoperative morbidity, including overall complications, unplanned ICU admissions, reinterventions, and hospital readmissions. These associations remained consistent after adjustment for relevant clinical confounders, supporting the robustness of the observed effect of ERAS pathways in elderly patients undergoing high-complexity colorectal surgery. Although ERAS was not significantly associated with 30 day readmissions in unadjusted analyses, this relationship became significant after adjustment for baseline and perioperative differences, suggesting that confounding in crude comparisons may have masked a protective effect.

ERAS pathways may be particularly effective in elderly patients because they directly target key age-related vulnerabilities, including limited physiological reserve, susceptibility to immobility-related complications, and impaired functional recovery. Early mobilization, a core ERAS element, has been shown to reduce the risk of postoperative complications and accelerate the recovery of functional walking capacity and other daily activities, which are essential for preserving autonomy in older adults^[^20^]^. ERAS protocols also integrate strategies such as multimodal analgesia, early oral intake, and stress reduction to support a faster return to baseline function, an effect that is particularly valuable in patients with limited physiological reserve^[^21,22^]^. Additionally, structured exercise and prehabilitation components within ERAS improve muscle strength, cardiorespiratory fitness, and overall physiological resilience, further enhancing postoperative functional recovery in high-risk populations^[^23^]^.

Our findings are consistent with those reported by Bagnall *et al* in 2014, who conducted a systematic review evaluating ERAS protocols in elderly patients undergoing colorectal surgery and concluded that ERAS is both feasible and effective in this population^[^24^]^. Across the 16 included studies, elderly patients managed with ERAS consistently demonstrated shorter hospital length of stay and lower postoperative complication rates compared with those receiving conventional care, without increases in mortality or readmission rates. Notably, none of the studies in that review included Latin American populations. In this context, the overall ERAS adherence rate of 80% observed in our cohort further supports the feasibility of protocol implementation in older adults and provides real-world evidence from a Latin American, middle-income setting, where data on ERAS outcomes in elderly patients undergoing complex colorectal surgery remain scarce.

The overall postoperative complication rate observed in our cohort was 38.5%. However, direct comparisons should be interpreted cautiously, as most regional studies have evaluated heterogeneous adult populations rather than elderly-specific cohorts. Previously published ERAS experiences in the region have reported overall postoperative complication rates of around 35%, whereas the ERAS group in our elderly cohort demonstrated a lower complication rate of 26.6% despite including exclusively older adults undergoing high-complexity colorectal surgery^[^25^]^. These findings highlight the potential benefit and feasibility of ERAS pathways in elderly patients, a subgroup that remains underrepresented in Latin American colorectal surgery research.

Although the study included a historical control cohort from 2018 onward, the institutional surgical and perioperative standards remained stable throughout the study period. One of the colorectal surgeons had implemented an ambulatory colorectal surgery protocol in 2014, which was subsequently adapted following ERAS implementation. From 2022 onward, all patients managed under this pathway received perioperative care according to ERAS Society recommendations, with protocol modifications and updates incorporated over time. In this context, the observed difference in active smoking prevalence between groups may partially reflect the effect of the ERAS pathway itself, as preoperative smoking cessation is actively encouraged as part of perioperative optimization, in addition to potential differences in documentation practices over time.

Several limitations should be acknowledged. The historical control design introduces potential temporal bias and unmeasured confounding; however, surgical complexity was comparable between groups, and multivariable adjustment accounted for relevant clinical variables. Although patients older than 80 years were included in both study groups, an exploratory subgroup analysis in this very elderly population was considered; however, given the limited sample size (20 patients in the non-ERAS group and 26 in the ERAS group), performing adjusted analyses and drawing robust conclusions would not have been methodologically appropriate. Finally, frailty was not formally assessed using a validated instrument, limiting physiological risk stratification beyond chronological age.

In conclusion, ERAS implementation among elderly patients undergoing high-complexity colorectal surgery was associated with a shorter hospital length of stay and lower postoperative morbidity, as well as fewer ICU admissions, reinterventions, and readmissions. These findings support the feasibility and effectiveness of ERAS pathways in older adults and provide important real-world evidence from a Latin American, middle-income setting, where data remain limited.

## Data Availability

The data that support the findings of this study are not publicly available due to institutional privacy and confidentiality restrictions. De-identified data may be available from the corresponding author upon reasonable request and with permission from the institutional ethics committee.

## Research registration unique identifying number (UIN)

Not applicable. This was a retrospective cohort study based on institutional medical records and did not involve prospective patient enrolment.
